# Sterilization Effects of Adult-targeted Baits Containing Insect Growth Regulators on *Delia antiqua*

**DOI:** 10.1038/srep32855

**Published:** 2016-09-13

**Authors:** Fangyuan Zhou, Guodong Zhu, Haipeng Zhao, Zheng Wang, Ming Xue, Xianxian Li, Huaqiang Xu, Xiaodan Ma, Yanyan Liu

**Affiliations:** 1 College of Plant Protection, Shandong Agricultural University, Shandong Province, 271000, China; 2 Non-point Source Pollution Remediation Laboratory, Ecology Institute, Shandong Academy of Sciences, Shandong Province, 250014, China; 3 Shandong Provincial Key Laboratory of Applied Microbiology, Shandong Academy of Sciences, Shandong Province, 250014, China; 4 Taierzhuang Agriculture Bureau Zaozhuang City, Shandong Province, 277400, China; 5 Shandong Sino-Agri United Biotechnology Co.,Ltd, Shandong Province, 250014, China; 6 Shenzhen Noposion Agrochemicals Co., Ltd, Shenzheng Province, 518102, China.

## Abstract

The onion maggot, *Delia antiqua*, is a devastating pest of liliaceous crops and current control measures fail to avert pesticide residues, threats to agroecosystem, and costly expenditures. Insect growth regulators (IGRs) are used as trypetid pest chemosterilants for their suppression on adult fertility and fecundity, but their effects on onion flies are unknown. Here, three IGRs (lufenuron, cyromazine, pyriproxyfen) were incorporated into baits to evaluate their effects on onion fly survival, fecundity, fertility, susceptibility of adults in different ages and offspring development. Lufenuron and cyromazine did not affect survival of new-emerged adults, but lufenuron inhibited adult fertility without affecting fecundity, and cyromazine reduced fertility and fecundity. Differently, pyriproxyfen enhanced fecundity within 10 days after treatment, while it reduced adult survival without affecting fertility. The fertility of younger adults was affected by lufenuron and cyromazine whereas the fecundity was affected with cyromazine and pyriproxyfen. For offspring of onion flies treated with lufenuron or cyromazine, most of larvae died within 5 days after hatch, but surviving larvae pupated and emerged normally. Pyriproxyfen did not affect offspring larval survival or pupation but affected pupal emergence. Thus, lufenuron and cyromazine could be potential chemosterilants for onion flies.

*Delia antiqua* (Meigen) (Diptera: Anthomyiidae) is a devastating crop pest distributed widely in many temperate countries such as Canada, Mexico, United States, China and Japan^1^. Its larva, onion maggot, feeds on bulb onions, garlics as well as other liliaceous crops, which leads to rot of the damaged parts^2^. If not controlled, it can reduce onion yield by as much as 80%^3^,^4^ and lead to garlic economic loss up to 50–70%^5^.

As the crop damage is directly caused by onion maggot feeding, present control methods are mainly targeted at the larva. Common methods for larval control are insecticides application in the furrow at planting^6^, seed coating treatments for onions and application of pesticides into irrigation water in garlic and onion fields^4^,^6^,^7^,^8^. The most widely used insecticides are organophosphates, carbamates and neonicotinoids^6^,^9^,^10^. Other methods targeting onion flies (adults) include spraying pesticides on crop leaves^11^, bait trapping stations^12^,^13^,^14^,^15^, repelling female oviposition with plastic strips^16^ and the male sterile technique^17^. However, application of chemicals to the crop results in pesticide contamination, which may cause food poisoning. Besides, large scale applications of chemical pesticides in the field could pose a great threat to agroecological environments^18^. In addition, repelling female oviposition with plastic strips and the male sterile technique cost too much. Thus, new management methods have to be developed as an alternative to these traditional ones for onion maggot control.

Recently, a group of chemicals called insect growth regulators (IGRs) have been used in baits to control trypetid and some dipteran pests as these chemicals inhibit adult reproduction^19^,^20^,^21^,^22^. Those chemicals have been incorporated into baits, fed to dipteran pests and shown excellent sterilizing effects on dipteran adults. For example, lufenuron reduces fertility of the Mexican fruit fly (*Anastrepha ludens*), the West Indian fruit fly (*A. oblique*), the sapote fruit fly (*A. serpentine*), the guava fruit fly (*A. striata*), the Mediterranean fruit fly (*Ceratitis capitata*), *Bactrocera dorsalis,* the melon fly (*B. cucurbitae*), the olive fruit fly (*B. oleae*) and the solanum fruit fly (*B. latifrons*) when ingested by adults^19^,^20^,^23^. Cyromazine also leads to sterility when ingested by adults of *C. capitata*^24^,^25^, *Musca domestica*^22^,^26^, and *Lucilia cuprina*^27^. In addition, lufenuron suppressed *C. capitata* population in field experiments^28^,^29^,^30^,^31^,^32^, and pyriproxyfen has been used to manage tsetse fly^33^,^34^,^35^ as well as *Haematobia irritans*^36^ in the field too. However, those studies mainly focus on the effects of IGRs on fertility and fecundity, while other parameters such as adult survival and offspring development are scarcely studied.

The onion fly adult needs to feed after emergence to meet the requirement of reproductive system development^37^, which suggests that baits containing pesticides could be used in onion maggot management. Given that IGRs showed excellent sterilization effects on dipteran pests, these chemicals might also be active against onion flies. The aim of this work is to investigate the effect of several selected IGRs used in baits on onion fly adult survival, fecundity, fertility, susceptibility of adults in different ages and offspring development, which would provide support information for them being used as onion fly chemosterilants. With lufenuron, pyriproxyfen and cyromazine selected as representative IGRs, this study also tried to compare the effect of IGRs with different modes of action on onion fly reproduction. Lufenuron is a benzoylurea chitin-synthesis inhibitor which interferes with the deposition of new cuticle during moulting^38^. Pyriproxyfen is a juvenile hormone mimic which prevents maturation of larvae to adults^39^. Cyromazine is a chitin-synthesis inhibitor with supposed ecdysone activity^40^,^41^.

## Results

### Survival, fecundity and fertility when 1-day old onion flies were fed with different doses of lufenuron, pyriproxyfen and cyromazine

Survival of the onion fly was not affected by the 72 h treatment of lufenuron at doses of 100, 500 or 1000 mg kg^−1^ during the 30-day period (Table 1). Differently, survival of onion flies treated by pyriproxyfen at doses of 100, 500 or 1000 mg kg^−1^ showed no significant difference compared to control on day 5 and 10 after emergence. The survival decreased significantly and was directly related to pyriproxyfen dose compared to the control on day 15, 20, 25 and 30 after emergence (Table 1). For cyromazine, the 72 h treatment at doses of 100, 500 and 1000 mg kg^−1^ did not affect survival compared to the control at each time point during the experimental period (Table 1).

Fecundity of onion flies was not affected by lufenuron at doses of 100, 500 and 1000 mg kg^−1^ compared to the control at each time point (Table 2). Pyriproxyfen showed multiple effects on onion fly fecundity. Particularly, fecundity of the onion fly on 10 day after emergence was stimulated significantly to 32.72 ± 9.73 and 34.14 ± 12.04 by pyriproxyfen at doses of 100 and 500 mg kg^−1^, respectively, which was significantly higher than that of the control (18.72 ± 4.19), while 1000 mg kg^−1^ of this IGR did not affect fecundity (Table 2). Fecundity on day 15 after emergence was not affected by pyriproxyfen at doses of 100 and 500 mg kg^−1^, while 1000 mg kg^−1^ reduced fecundity significantly (Table 2). Effects of pyriproxyfen on onion fly fecundity were similar on day 20, 25 and 30 after emergence (Table 2), i.e., fecundity decreased significantly in response to increasing pyriproxyfen doses compared to the control. Onion fly fecundity was significantly decreased by the 72 h treatment of cyromazine at doses of 100, 500 and 1000 mg kg^−1^, and it was directly related to cyromazine doses compared to the control (Table 2). On day 30 after emergence, fecundity of cyromazine-treated onion fly was reduced to 116.40 ± 7.55, 94.48 ± 19.26, 74.04 ± 14.74 at doses of 100, 500 and 1000 mg kg^−1^, respectively, which was significantly lower than that of the control (164.10 ± 15.40).

The onion fly fertility was significantly decreased by lufenuron at doses of 100, 500 and 1000 mg kg^−1^, and it was directly related to doses compared to the control till day 25 after emergence (Table 3). At a dose of 1000 mg kg^−1^, lufenuron reduced fertility to 13.09 ± 4.36%, 16.19 ± 2.61%, 27.61 ± 2.33%, 38.57 ± 3.61% and 60.57 ± 2.71% on day 5, 10, 15, 20, 25, and 30, respectively, which were all significantly lower than that of the control. On the contrary, pyriproxyfen treatments showed no effects on fertility of the onion fly at each time point (Table 3). Compared to the control, cyromazine significantly decreased the fertility of onion flies on day 5 and 10 after emergence, while these inhibition effects disappeared on day 15, 20, 25, and 30 after emergence (Table 3).

### Fecundity and fertility of onion flies which were treated in different ages by lufenuron, pyriproxyfen and cyromazine

Fecundity of 1-day, 4-day and 7-day old onion flies treated with 0, 50 or 500 mg kg^−1^ of lufenuron was not significantly different with each other (Table 4). Pyriproxyfen decreased fecundity of onion fly adults treated in different ages. Specifically, under the treatment of pyriproxyfen, the onion fly fecundity decreased significantly as the treated adult age decreased (Table 4). Fecundity of 7-day, 4-day and 1-day old onion flies when treated with pyriproxyfen at doses of 50 mg kg^−1^ was 135.84 ± 14.53, 115.48 ± 7.82 and 89.23 ± 7.87, respectively. With the treatment of pyriproxyfen at doses of 500 mg kg^−1^, fecundity of 7-day, 4-day and 1-day old onion flies was 136.92 ± 11.24, 95.87 ± 9.58 and 67.95 ± 7.32, respectively. Similar with pyriproxyfen, inhibition effects of cyromazine on onion fly fecundity decreased significantly as the treated adult age increased (Table 4). For onion flies treated by cyromazine at a dose of 50 mg kg^−1^, fecundity of 1-day, 4-day and 7-day old onion flies was 95.75 ± 11.19, 149.98 ± 13.42 and 153.63 ± 18.99, respectively. For onion flies treated by cyromazine at a dose of 500 mg kg^−1^, fecundity of 1-day, 4-day and 7-day old onion flies was 64.02 ± 13.68, 102.87 ± 7.85 and 150.77 ± 14.10, respectively.

As it was shown in Table 5, fertility of 1-day, 4-day and 7-day old onion flies fed with baits containing lufenuron was significantly different with each other, and fertility of treated onion fly decreased significantly as the adult age increased. For onion flies treated by 50 mg kg^−1^ of lufenuron, fertility of 1-day, 4-day and 7-day old adult flies was 79.29 ± 4.46%, 66.14 ± 4.67% and 49.71 ± 5.53%, respectively. For onion flies treated with 500 mg kg^−1^ of lufenuron, fertility of 1-day, 4-day and 7-day old adult flies was 36.86 ± 5.21%, 18.43 ± 4.61% and 5.14 ± 5.18%, respectively. Under the treatment of pyriproxyfen, fertility of 1-day, 4-day and 7-day old onion flies was not significantly different with each other (Table 5). Inhibition effects of cyromazine on onion flies increased significantly as adult age increased (Table 5). Fertility of 7-day old onion fly was decreased to 8.57 ± 6.27% by the 72 h treatment of cyromazine at a dose of 500 mg kg^−1^, which was significantly lower than that of 4-day old (32.29 ± 6.16%) and 1-day old (58.57 ± 6.73%) onion flies.

### Offspring larval development of onion flies treated by lufenuron, pyriproxyfen and cyromazine

Offspring larval survival on day 5 after hatch of 50 mg kg^−1^ lufenuron-treated onion flies was significantly reduced to 55.00 ± 11.93%, which was lower than that of the control (84.29 ± 8.38%, Table 6). Pyriproxyfen did not affect offspring larval survival on day 5 after hatch when parent flies were treated at a dose of 50 mg kg^−1^ (Table 6). Cyromazine reduced offspring larval survival on day 5 after hatch to 78.86 ± 6.64% when parent flies were fed with 50 mg kg^−1^ of cyromazine, which was significantly lower than that of the control (92.00 ± 6.93%, Table 6).

Offspring larval survival on day 15 after hatch was not affected significantly when parent flies were fed with sugar baits containing 50 mg kg^−1^ of lufenuron, pyriproxyfen or cyromazine, respectively (Table 6).

Offspring pupation rate was not affected significantly when parent flies were fed with sugar baits containing 50 mg kg^−1^ of lufenuron, pyriproxyfen or cyromazine, respectively (Table 6).

Emergence rate of offspring pupae when parent flies were fed with sugar baits containing 50 mg kg^−1^ of lufenuron or cyromazine was not affected compared to the control (Table 6). However, it was significantly reduced to 63.57 ± 7.48% with pyriproxyfen, which was significantly lower than that of the control (97.14 ± 7.56%).

## Discussion

Although insect growth regulators have been used as chemosterilants for fruit flies^19^,^20^,^22^,^23^,^26^,^28^,^29^,^30^,^31^,^32^, this is the first laboratory attempt to use IGRs in adult-targeted baits to control onion maggot damage. In this study, IGRs with different modes of action were chosen to represent 3 kinds of the most widely used IGRs insecticides at present. In addition, detailed life parameters of the onion fly including adult survival, fecundity, fertility, susceptibility of adult in different ages and offspring development were observed, which would provide sufficient justification for IGRs being used as chemosterilants. Results in this study showed that these chemicals in sugar baits inhibited onion fly reproduction in various ways.

The most remarkable effect of lufenuron on onion flies was the significant inhibition of fertility when adult flies were fed it (Tables 3 and 5). Similar effects had been reported on *C. capitata*^23^,^42^, *A. ludens*^20^, *A. obliqua*^20^, *A. serpentina*^20^, *A. striata*^20^, *B. dorsalis*^23^*, B. latifron*^23^, *M. domestica*^43^ and *B. oleae*^19^. In this study, the hooked mouth part could be observed with stereoscopic microscopes in unhatched eggs produced by lufenuron-treated onion flies, which indicated that the embryo had developed completely even though the larva failed to get out of the egg shell (Supplementary, Fig. S1). Lufenuron had been reported to interfere with the deposition of new cuticle in insects^38^. Thus, the developed embryo cuticle containing chitin maybe affected by lufenuron, being less rigid, is unable to act as an effective exoskeleton for the attached musculature. This effect was similar to another benzoylphenyl urea chitin-synthesis inhibitor diflubenzuron which decreased fertility of the housefly^44^. Another interesting aspect was the effect of lufenuron and the age of adult flies on fertility. Results showed that older adult onion flies were more susceptible to IGRs resulting in decreased fertility (Table 5). Similar effects had been reported on *C. capitata*^42^ and *M. domestica*^43^,^45^. This effect may result from incorporation of relatively more lufenuron into forming eggs in older adults, while these chemicals might have been metabolized and excreted before they were incorporated into eggs in younger adult females. Besides, most of offspring larvae died within 5 days of hatching when parent onion flies were treated by lufenuron, which was of great significance in crop protection as less new-born larvae could develop to third instar that are responsible for severe damage to crops.

Pyriproxyfen, a juvenile hormone analogue, shortened preoviposition period of onion flies when treated at a dose of 100 mg kg^−1^, while higher doses did not affect preoviposition period (Supplementary, Table S2). What’s more interesting was that it enhanced egg production during short period after non-lethal dose of treatments (Table 2). This is consistent with another species. A non-lethal dose of pyriproxyfen enhances the fecundity significantly as well as the ovarian development when *Rhagoletis pomonella* adults are treated by topical application^46^. Former researchers discussed this enhanced fecundity as hormesis^19^, while it is more like juvenile hormone regulation. Contrary to increased fecundity, survival of adult female was reduced significantly (Table 1). This may be energy compromise between survival and reproduction in a short period. Pyriproxyfen had no effects on fertility when 1-day old onion fly adult females were treated (Table 5). Fertility of matured *Stomoxys calcitrans*^47^, *B. oleae*^19^, and *R. pomonella*^46^ adult is not affected when treated by pyriproxyfen, while fertility of unmatured *S. calcitrans*^47^, *C. capitata*^42^ was reduced significantly. In this study, it seemed that fertility of older onion fly females was not affected by pyriproxyfen (Table 5). Although survival had been significantly reduced in this experiment and former researchers proved that pupae produced by pyriproxyfen treated female adult cannot emerge^36^,^42^,^48^, pyriproxyfen cannot be used in the field as farmers cannot bear potential serious damage caused by numerous larvae hatched from increased fecundity.

The most impressive effect of cyromazine on onion flies was the sharply decreased fecundity of 1-day old onion flies (Table 2). Besides, the younger treated adults were less fecund, whereas the fecundity of matured onion flies was not affected (Table 4). Similar effects had also been reported on *L. sericata*^27^,^49^,^50^, *A. ludens*^21^, *B. oleae*^19^, *C. capitata*^24^,^25^ and *Drosophila melanogaster*^51^. Inhibitory effects of this chemical on fecundity of dipteran insects may depend on the age of females when they are treated, and fecundity of unmatured adult female are more likely to be affected. The average preoviposition period of onion fly female is 7 days^2^, and fecundity of 7-day old adult females was not affected (Table 4). As for the reduced fecundity, there may be three reasons. First, cyromazine inhibited development of the reproduction system, especially the ovaries. Cyromazine prolonged the preoviposition period of newly emerged onion flies (Supplementary, Table S2), which indicated that cyromazine may inhibit vitellogenesis in the ovary. Second, eggs may developed normally in ovaries, but they could not be laid. This may result from reduced mechanical strength of onion fly female ovipositor, which had also been reported on *A. ludens*^52^. Mechanical strength of unmatured *A. ludens* female ovipositor is affected by cyromazine^52^, while its ovary development is not affected directly by cyromazine^21^,^24^. Third, eggs formed normally in female ovaries, but they were resorbed before being laid. Cyromazine is of ecdysone activity, and it has been hypothesized to be related to the development of hormone 20-hydroxyecdysone^40^,^41^. Low concentrations of ecdysteroid are essential for normal oogenesis, while there is a threshold concentration in egg chambers and that apoptosis at mid-oogenesis is induced when the ecdysteroid levels exceed that threshold^53^. Fruther researches need to be conducted to define mechanisms of cyromazine inhibition on fecundity.

Considering lufenuron, pyriproxyfen and cyromazine as representative chemicals, this research not only determined the effects of these 3 IGRs on onion fly reproduction but also provides support for predicting effects of IGRs with similar modes of action on onion fly reproduction. Thus, combined applications of these selected IGRs on onion flies were not conducted although it could show more significant inhibition effects on reproduction. Overall, lufenuron and cyromazine affected onion fly reproduction when these chemicals were fed to flies, which indicated that they could be used as chemosterilants for onion flies. Besides, these two chemicals didn’t affect survival of onion flies indicating that they could be of great advantages in conservation of natural enemy especially those predatory adults. Our results indicate that IGRs with similar modes of actions could be potential chemosterilants for onion fly control. There would be many advantages if these chemicals were used as chemosterilants for onion flies. First, as IGRs specifically interfere with chitin deposition which was only discovered in insect cuticle or work as specific hormones influence insect maturity and reproduction mediation^54^, they are of great safety to mammal and human as IGRs act specifically on arthropods. This makes them optimized chemicals to be used in baits for onion flies. Second, IGRs used in baits combined with specific attractants could avoid application of pesticides in large scale such as foliar spraying and reduce environmental impact especially on non-targeted beneficial insects. Third, some of these IGRs such as lufenuron and cyromazine could kill offspring larvae before them making threats to crops, which is of great significance in practical crop protection. In this study, inhibitory effects of IGRs on onion fly males or females were not detected respectively as we mainly focused on their practical performance in the field. As for IGRs effects on onion fly male or female, further experiments shall be conducted to illustrate mechanical details of these inhibitory effects on reproduction. Although further work have to be conducted to test their control efficiency in the field, this work may provide new options for onion maggot control.

## Methods

### Experiment I Effects of adult-ingested lufenuron, pyriproxyfen and cyromazine on onion fly survival, fecundity and fertility

Preliminary tests showed that the LC_50_ of lufenuron, pyriproxyfen and cyromazine was more than 2000 mg kg^−1^ at 72 h (Supplementary, Table S1). In case of unexpected onion fly death, lower treatment doses (100, 500 and 1000 mg kg^−1^) of these three IGRs insecticides were used to treat onion flies (emerged within 6 h) as described previously^20^,^55^. Detailed information were in supplementary methods online. Briefly, granulated sugar was impregnated with acetone or methanol-water solution containing different amount of insecticides, and the solvent was evaporated to obtain 100, 500, 1000 mg kg^−1^ doses of IGRs-sugar baits. Five pairs of flies were put into a cylinder-shaped glass chamber (open at both ends, L = 12 cm, ∅≈6 cm, supplementary, Fig. S2) and fed only with these IGRs-sugar baits and water for 72 h, and then these baits were replaced with milk and 5% sucrose water solution. Granulated sugar was used as control as acetone or methanol impregnation showed no effects on onion flies (Data not shown). All flies survived from the 72-hour treatment due to the low toxicity of the selected IGRs. Each group of treated onion flies (in each glass chamber) was regarded as one replication, and each treatment group was replicated 7 times.

To test effects of these selected IGRs on survival of onion flies, the number of remaining alive adults in each chamber was recorded every 24 h. Survival of onion flies in each glass chamber (each replication) at 5, 10, 15, 20, 25, and 30 d after eclosion was calculated.

To study the effect of these selected IGRs on fecundity of onion flies, eggs laid during last 24 h by females from each chamber were collected and counted every day from first oviposition up to the 30th day after eclosion. In order to obtain the quantity of eggs laid per female per day, the total number of eggs from one chamber was divided by the number of remaining females yesterday in that chamber. Fecundity, i.e., the quantity of laid eggs accumulated at 5, 10, 15, 20, 25, and 30 d after eclosion per female in each glass chamber (one replication) was calculated.

To study the effect of adult-ingested IGRs on fertility (larval hatch), 30 eggs from each chamber were sampled randomly at 5, 10, 15, 20, 25, and 30 d after treatment and placed in plastic petri dishes containing a round piece of permanently wet filter paper, covered with a black humid cloth, and maintained at 21 ± 0.5 °C and 70–75% RH. Egg hatch was checked using an Olympus stereoscopic microscope (Olympus Corporation, Japan) after 4 days of incubation (The egg period of onion flies usually lasts about 3 days). Empty egg shells were regarded as hatched larvae, and hatch percent of these 30 eggs from each glass chamber (one replication) was calculated.

### Experiment II Effects of adult-ingested lufenuron, pyriproxyfen and cyromazine on fecundity and fertility of adults treated in different ages

Onion fly adults in different ages (different maturation stages) may respond differently to IGRs treatments. The average preoviposition period of the onion fly is 7 days^2^. Thus, 1-day old (emerged within 6 h, undeveloped reproduction system), 4-day old (emerged about 96 h, developing reproduction system) and 7-day old (emerged about 168 h, developed reproduction system) adult onion flies which had been starved for 24 h (for 4-day and 7-day old onion flies) were fed with sugar baits containing 50 and 500 mg kg^−1^ of lufenuron, pyriproxyfen and cyromazine. Granulated sugar was used as control. Five pairs of onion flies were put inside a glass chamber and treated, and each group of treated onion flies (in each glass chamber) was regarded as one replication. Each treatment was replicated 7 times. Detailed onion fly treatment procedure was in Experiment I and supplementary materials.

Onion flies survived from treatments and egg quantity in each glass chamber were checked and recorded every day, and fecundity at 30 d after emergence per female in each glass chamber (one replication) was calculated as described above.

To compare the response of onion fly fertility to selected IGRs when onion flies were treated in different developmental stages, 30 eggs from each chamber (each replication) were randomly collected at day 10 after treatment. The hatch percent (fertility) of these collected eggs was calculated as describe in Experiment I.

### Experiment III Effects of adult-ingested lufenuron, pyriproxyfen and cyromazine on offspring larval development

Adults in different developmental stages which showed the greatest susceptibility to IGRs according to the former experiment were used in this experiment. Sugar baits containing 50 mg kg^−1^ of IGRs were used to obtain sufficient hatched larvae for further observation. In this experiment, onion flies were treated with the similar method as described before. Adults were put in a cage (25 × 25 × 25 cm wood-profile cages) instead of in a glass chamber. Specifically, twenty pairs of onion flies were put inside a cage and treated, and was regarded as one replication (in each cage). Granulated sugar was used as control. Each treatment was replicated 7 times. Eight days after treatment, one hundred eggs from each cage were collected randomly and incubated as described above. Three pieces of garlic were put aside the eggs as food for hatched larvae, and the larval development from each cage was observed continuously until they pupated. For each group of eggs (from one cage), fertility was recorded after 4 days of incubation. Five-day old larvae after hatch are too small to make significant threats to crops, while 15-day old larvae damage crops. Thus, we tried to evaluate the larva survival at day 5 and 15 after egg hatch as follows:


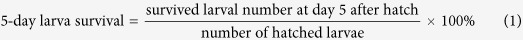



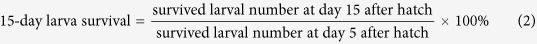


Besides, pupation rate and pupal emergence rate were also calculated as follows:


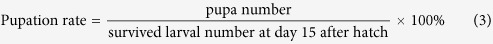



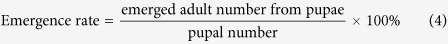


### Statistical analysis

Prior to statistical analysis we tested all variables for normality with the Kolmogorov-Smirnov test and homogeneity of group variances with Levene’s test. In Experiment I, onion fly survival, fecundity or fertility at each time point was regarded as variable, and IGRs doses were regarded as independent variables in one-way ANOVA and followed by Tukey’s HSD multiple comparisons. In Experiment II, fecundity or fertility of onion flies treated with each insecticide dose (including the 0 mg kg^−1^ group, i.e., the control group) was regarded as dependent variable, and onion fly developmental stages were regarded as independent variables in one-way ANOVA and followed by Tukey’s HSD multiple comparisons. For 5-day larval survival, 15-day larval survival, pupation rate, and emergence rate in Experiment III, data were compared by using an independent-samples *t* test. All analyses were performed with PASW Statistics 18.0.0 (2009; SPSS Inc. Quarry Bay, HK).

## Additional Information

**How to cite this article**: Zhou, F. *et al.* Sterilization Effects of Adult-targeted Baits Containing Insect Growth Regulators on *Delia antiqua. Sci. Rep.* **6**, 32855; doi: 10.1038/srep32855 (2016).

## Supplementary Material

Supplementary Information

## Figures and Tables

**Table 1:** Survival of onion flies treated by selected IGRs.

IGRs	Time^a^	Dose (mg kg^−1^)				*F*	df	P
		Control	100	500	1000			
Lufenuron	5	98.57 ± 3.78a	98.57 ± 3.78a	95.71 ± 7.87a	98.57 ± 3.78a	0.545	(3, 24)	0.656
	10	98.57 ± 3.78a	97.14 ± 4.88a	95.71 ± 7.87a	97.14 ± 4.88a	0.308	(3, 24)	0.820
	15	97.14 ± 4.88a	97.14 ± 4.88a	95.71 ± 7.87a	95.71 ± 5.35a	0.138	(3, 24)	0.936
	20	95.71 ± 5.35a	94.28 ± 7.87a	94.28 ± 7.87a	92.85 ± 7.56a	0.182	(3, 24)	0.908
	25	90.00 ± 8.16a	91.42 ± 6.90a	88.57 ± 6.90a	84.28 ± 12.72a	0.824	(3, 24)	0.494
	30	84.28 ± 5.35a	85.71 ± 12.72a	77.14 ± 12.53a	75.71 ± 12.72a	1.383	(3, 24)	0.272
Pyriproxyfen	5	98.57 ± 3.78a	98.57 ± 3.78a	98.57 ± 3.78a	98.57 ± 3.78a	0.333	(3, 24)	0.801
	10	95.71 ± 7.87a	92.85 ± 4.88a	91.42 ± 9.00a	90.00 ± 5.77a	0.833	(3, 24)	0.489
	15	90.00 ± 10.00a	72.85 ± 7.56b	67.14 ± 14.96bc	52.85 ± 7.56c	15.087	(3, 24)	<0.01
	20	90.00 ± 10.00a	67.14 ± 9.51b	48.57 ± 6.90c	25.71 ± 5.35d	78.357	(3, 24)	<0.01
	25	87.14 ± 9.51a	57.14 ± 9.51b	35.71 ± 13.97c	17.14 ± 9.51d	54.245	(3, 24)	<0.01
	30	77.14 ± 7.56a	25.71 ± 9.76b	11.42 ± 10.69c	4.29 ± 7.87c	92.217	(3, 24)	<0.01
Cyromazine	5	98.57 ± 3.78a	97.14 ± 7.56a	97.14 ± 7.56a	95.71 ± 7.87a	0.200	(3, 24)	0.895
	10	97.14 ± 4.88a	94.28 ± 9.76a	94.28 ± 11.33a	94.28 ± 7.87a	0.185	(3, 24)	0.906
	15	94.28 ± 7.87a	92.85 ± 9.51a	91.42 ± 12.14a	90.00 ± 11.54a	0.220	(3, 24)	0.882
	20	88.57 ± 12.14a	85.71 ± 13.97a	85.71 ± 13.97a	81.42 ± 15.73a	0.309	(3, 24)	0.819
	25	85.71 ± 9.76a	82.85 ± 12.53a	80.00 ± 11.54a	78.57 ± 13.45a	0.496	(3, 24)	0.689
	30	82.85 ± 7.56a	77.14 ± 7.56a	75.71 ± 12.72a	72.85 ± 12.53a	1.143	(3, 24)	0.352

Within each row in the table above, values (survival, %, mean ± s.e., n = 7) were analyzed with ANOVA, and different letters denote significant differences (Tukey’s HSD test, p < 0.05). ^“a”^means the day after emergence.

**Table 2:** Fecundity of onion flies treated by selected IGRs.

IGRs	Time^a^	Dose (mg kg^−1^)				*F*	df	P
		Control	100	500	1000			
Lufenuron	5	−b	−	−	−	−	−	−
	10	13.00 ± 4.62a	12.25 ± 3.43a	12.06 ± 3.07a	15.28 ± 5.07a	0.898	(3, 24)	0.457
	15	53.37 ± 8.27a	46.46 ± 6.56a	46.80 ± 7.36a	54.37 ± 7.19a	2.277	(3, 24)	0.105
	20	77.61 ± 11.77a	70.40 ± 8.78a	73.85 ± 8.52a	76.61 ± 14.57a	0.584	(3, 24)	0.631
	25	107.00 ± 21.41a	100.10 ± 14.83a	97.75 ± 15.02a	105.50 ± 23.13a	0.371	(3, 24)	0.774
	30	132.40 ± 28.07a	114.20 ± 13.57a	116.00 ± 15.79a	122.30 ± 27.47a	0.952	(3, 24)	0.431
Pyriproxyfen	5	−	−	−	−	−	−	−
	10	18.72 ± 4.19b	32.72 ± 9.73a	34.14 ± 12.04a	15.80 ± 3.55b	9.216	(3, 24)	<0.01
	15	50.82 ± 9.22a	60.95 ± 8.04a	49.52 ± 8.29a	29.22 ± 10.30b	15.244	(3, 24)	<0.01
	20	88.27 ± 9.74a	84.72 ± 11.82a	58.04 ± 15.41b	35.90 ± 6.47c	32.910	(3, 24)	<0.01
	25	120.50 ± 13.79a	113.40 ± 14.09a	61.58 ± 9.18b	36.78 ± 10.85c	77.868	(3, 24)	<0.01
	30	155.80 ± 14.71a	121.20 ± 14.67b	64.08 ± 9.17c	45.54 ± 9.92d	118.173	(3, 24)	<0.01
Cyromazine	5	−	−	−	−	−	−	−
	10	19.81 ± 3.00a	10.48 ± 3.47b	8.83 ± 2.79b	2.64 ± 1.40c	45.840	(3.24)	<0.01
	15	69.65 ± 12.61a	42.15 ± 10.44b	39.13 ± 7.48b	19.89 ± 5.13c	33.565	(3, 24)	<0.01
	20	104.70 ± 15.44a	79.33 ± 8.50b	74.90 ± 16.28b	36.89 ± 13.29c	29.157	(3, 24)	<0.01
	25	133.30 ± 21.81a	106.80 ± 12.16b	95.09 ± 20.10b	63.38 ± 17.51c	17.651	(3, 24)	<0.01
	30	164.10 ± 15.40a	116.40 ± 7.55b	94.48 ± 19.26c	74.04 ± 14.74c	47.412	(3, 24)	<0.01

Within each row in the table above, values (fecundity, mean ± s.e., n = 7) were analyzed with ANOVA, and different letters denoted significant differences (Tukey’s HSD test, p < 0.05). ^“a”^means the day after emergence. ^“b”^mean onion flies did not oviposit on day 5 after emergence.

**Table 3:** Fertility of onion flies treated by selected IGRs.

IGRs	Time^a^	Dose (mg kg^−1^)				*F*	df	*p*
		Control	100	500	1000			
Lufenuron	5	98.57 ± 2.99a	36.99 ± 4.07b	23.85 ± 2.90c	13.09 ± 4.36d	762.228	(3, 24)	<0.01
	10	95.71 ± 4.60a	37.66 ± 4.73b	31.47 ± 5.28c	16.19 ± 2.61d	553.649	(3, 24)	<0.01
	15	95.71 ± 5.92a	59.28 ± 4.60b	43.33 ± 3.73c	27.61 ± 2.33d	307.798	(3, 24)	<0.01
	20	83.57 ± 3.61a	77.57 ± 6.05a	64.85 ± 6.55b	38.57 ± 3.61c	89.835	(3, 24)	<0.01
	25	85.14 ± 3.07a	85.42 ± 2.35a	80.47 ± 8.23a	60.57 ± 2.71b	42.184	(3, 24)	<0.01
	30	82.47 ± 5.10a	82.80 ± 5.33a	84.42 ± 2.21a	81.61 ± 10.20a	0.246	(3, 24)	0.864
Pyriproxyfen	5	92.19 ± 4.69a	93.66 ± 5.06a	93.85 ± 5.79a	90.47 ± 4.16a	0.664	(3, 24)	0.582
	10	85.00 ± 6.31a	88.90 ± 3.35a	85.00 ± 9.36a	84.14 ± 7.00a	0.654	(3, 24)	0.588
	15	91.42 ± 3.90a	87.47 ± 6.54a	86.14 ± 6.00a	86.76 ± 6.44a	1.153	(3, 24)	0.348
	20	86.90 ± 6.70a	81.47 ± 8.26a	85.66 ± 6.75a	86.00 ± 8.76a	0.669	(3, 24)	0.579
	25	90.00 ± 5.09a	83.85 ± 9.49a	86.76 ± 6.31a	88.57 ± 7.02a	0.885	(3, 24)	0.463
	30	87.14 ± 8.70a	83.80 ± 8.03a	80.95 ± 8.97a	86.66 ± 8.61a	0.757	(3, 24)	0.529
Cyromazine	5	90.47 ± 7.86a	82.33 ± 8.54a	31.85 ± 9.06b	9.76 ± 8.52c	148.406	(3, 24)	<0.01
	10	92.85 ± 6.36a	85.09 ± 8.20a	52.85 ± 8.46b	22.71 ± 9.55c	106.351	(3, 24)	<0.01
	15	89.80 ± 7.43a	92.38 ± 5.35a	91.52 ± 7.57a	89.42 ± 6.10a	0.320	(3, 24)	0.811
	20	85.95 ± 7.51a	87.61 ± 5.52a	84.09 ± 4.54a	85.00 ± 7.07a	0.424	(3, 24)	0.737
	25	83.80 ± 4.38a	85.57 ± 4.35a	84.19 ± 4.59a	85.04 ± 4.38a	0.238	(3, 24)	0.869
	30	85.04 ± 4.23a	84.52 ± 4.88a	85.47 ± 5.24a	85.00 ± 4.08a	0.057	(3, 24)	0.982

Within each row in the table above, values (fertility, %, mean ± s.e., n = 7) were analyzed with ANOVA, and different letters denote significant differences (Tukey’s HSD test, p < 0.05). ^“a”^means the day after emergence.

**Table 4:** Fecundity of 1-day, 4-day, and 7-day old onion flies when treated by selected IGRs.

Adult age	IGRs Dose (mg kg^−1^)								
	Lufenuron			Pyriproxyfen			Cyromazine		
	Control	50	500	Control	50	500	Control	50	500
1-day	121.54 ± 13.04a	121.33 ± 10.47a	123.19 ± 13.47a	133.73 ± 11.34a	89.23 ± 7.87c	67.95 ± 7.32c	178.07 ± 10.15a	95.75 ± 11.19b	64.02 ± 13.68c
4-day	125.55 ± 20.64a	124.11 ± 16.25a	124.92 ± 16.86a	135.37 ± 11.10a	115.48 ± 7.81b	95.87 ± 9.58b	180.63 ± 10.91a	149.48 ± 13.42a	102.87 ± 7.85b
7-day	125.04 ± 15.47a	123.08 ± 16.22a	112.95 ± 12.58a	131.01 ± 10.70a	135.84 ± 14.53a	136.92 ± 11.24a	176.44 ± 10.87a	153.63 ± 18.99a	150.77 ± 14.10a
*F*	0.119	0.065	1.409	0.279	34.222	93.017	0.275	32.856	88.635
df	2, 18	2, 18	2, 18	2, 18	2, 18	2, 18	2, 18	2, 18	2, 18
*p*	0.889	0.937	0.270	0.760	<0.001	<0.001	0.762	<0.001	<0.001

Within each column in the table above, values (fecundity, mean ± s.e., n = 7) were analyzed with ANOVA, and different letters denote significant differences (Tukey’s HSD test, p < 0.05).

**Table 5:** Fertility of 1-day, 4-day, and 7-day old onion flies when treated by selected IGRs.

Adult age	IGRs Dose (mg kg^−1^)								
	Lufenuron			Pyriproxyfen			Cyromazine		
	Control	50	500	Control	50	500	Control	50	500
1-day	94.29 ± 6.07a	79.29 ± 4.46a	36.86 ± 5.21a	92.14 ± 8.09a	90.54 ± 10.51a	89.29 ± 13.67a	96.43 ± 7.48a	91.86 ± 8.21a	58.57 ± 6.73a
4-day	95.00 ± 7.64a	66.14 ± 4.67b	18.43 ± 4.61b	97.14 ± 7.56a	92.86 ± 10.75a	92.73 ± 10.72a	95.71 ± 6.07a	73.00 ± 6.27b	32.29 ± 6.16b
7-day	94.29 ± 7.32a	49.71 ± 5.53c	5.14 ± 5.18c	91.43 ± 10.69a	95.00 ± 9.57a	92.86 ± 9.06a	94.29 ± 8.38a	55.71 ± 6.73c	8.57 ± 6.27c
*F*	0.024	63.773	70.798	0.440	0.329	0.224	0.153	45.133	107.258
df	2, 18	2, 18	2, 18	2, 18	2, 18	2, 18	2, 18	2, 18	2, 18
*p*	0.976	<0.001	<0.001	0.859	0.724	0.801	0.859	<0.001	<0.001

Within each column in the table above, values (fertility, %, mean ± s.e., n = 7) were analyzed with ANOVA, and different letters denote significant differences (Tukey’s HSD test, p < 0.05).

**Table 6:** Effects on larval development when onion fly adults were fed with IGRs.

%	IGRs	Dose (mg kg^−1^)		df	*t*	*p*
		Control	50			
5-day larval survival	Lufenuron	84.29 ± 8.38	55.00 ± 11.93*	12	5.314	<0.001
	Pyriproxyfen	85.29 ± 10.83	85.29 ± 8.75	12	−0.670	0.515
	Cyromazine	92.00 ± 6.93	78.86 ± 6.64*	12	3.622	0.003
15-day larval survival	Lufenuron	87.00 ± 6.58	87.71 ± 11.61	12	−0.142	0.890
	Pyriproxyfen	95.14 ± 8.47	91.57 ± 9.02	12	0.764	0.460
	Cyromazine	95.86 ± 4.10	94.43 ± 7.76	12	0.430	0.675
Pupation rate	Lufenuron	97.57 ± 4.24	97.14 ± 4.88	12	0.175	0.864
	Pyriproxyfen	97.14 ± 7.56	99.14 ± 2.27	12	−0.607	0.512
	Cyromazine	98.57 ± 3.78	97.14 ± 7.56	12	0.447	0.663
Emergence rate	Lufenuron	98.57 ± 3.78	97.14 ± 4.88	12	0.612	0.552
	Pyriproxyfen	97.14 ± 7.56	63.57 ± 7.48*	12	8.352	<0.001
	Cyromazine	97.14 ± 7.56	94.29 ± 7.82	12	0.693	0.502

Within each row in the table above, values (%, mean ± s.e., n = 7) were compared by using an independent-samples t test. “*” denotes significant differences (independent-samples t test, p < 0.05).
