# Supplementary material for: Sterilization Effects of Adult-targeted Baits Containing Insect Growth Regulators on Delia antiqua
Source: Sci Rep. 2016 Sep 13;6:32855. doi: 10.1038/srep32855 (PMC5020654; doi:10.1038/srep32855)
Supplement: Supplementary Information [file srep32855-s1.pdf]

Sterilization Effects of Adult-targeted Baits Containing Insect Growth Regulators on *Delia antiqua*

Fangyuan Zhou[1,2,3], Guodong Zhu[1], Haipeng Zhao[1], Zheng Wang[4], Ming Xue*[1], Xianxian Li[5], Huaqiang Xu[5], Xiaodan Ma[6], Yanyan Liu[1]

[1] College of Plant Protection, Shandong Agricultural University, Shandong Province, 271000, China.

[2] Non-point Source Pollution Remediation Laboratory, Ecology Institute, Shandong Academy of Sciences.

[3] Shandong Provincial Key Laboratory of Applied Microbiology, Shandong Academy of Sciences.

[4] Taierzhuang Agriculture Bureau Zaozhuang City, Shandong Province, 277400, China.

[5] Shandong Sino-Agri United Biotechnology Co.,Ltd.

[6] Shenzhen Noposion Agrochemicals Co., Ltd.

* Correspondence: *E-mail:* xueming@sdau.edu.cn

Supplementary:

Figures, tables and supplementary methods for "Sterilization Effects of Adult-targeted Baits Containing Insect Growth Regulators on *Delia antiqua*"

Figure S1 Embryo development in eggs when adult of *Delia antiqua* (Meigen) were treated with lufenuron

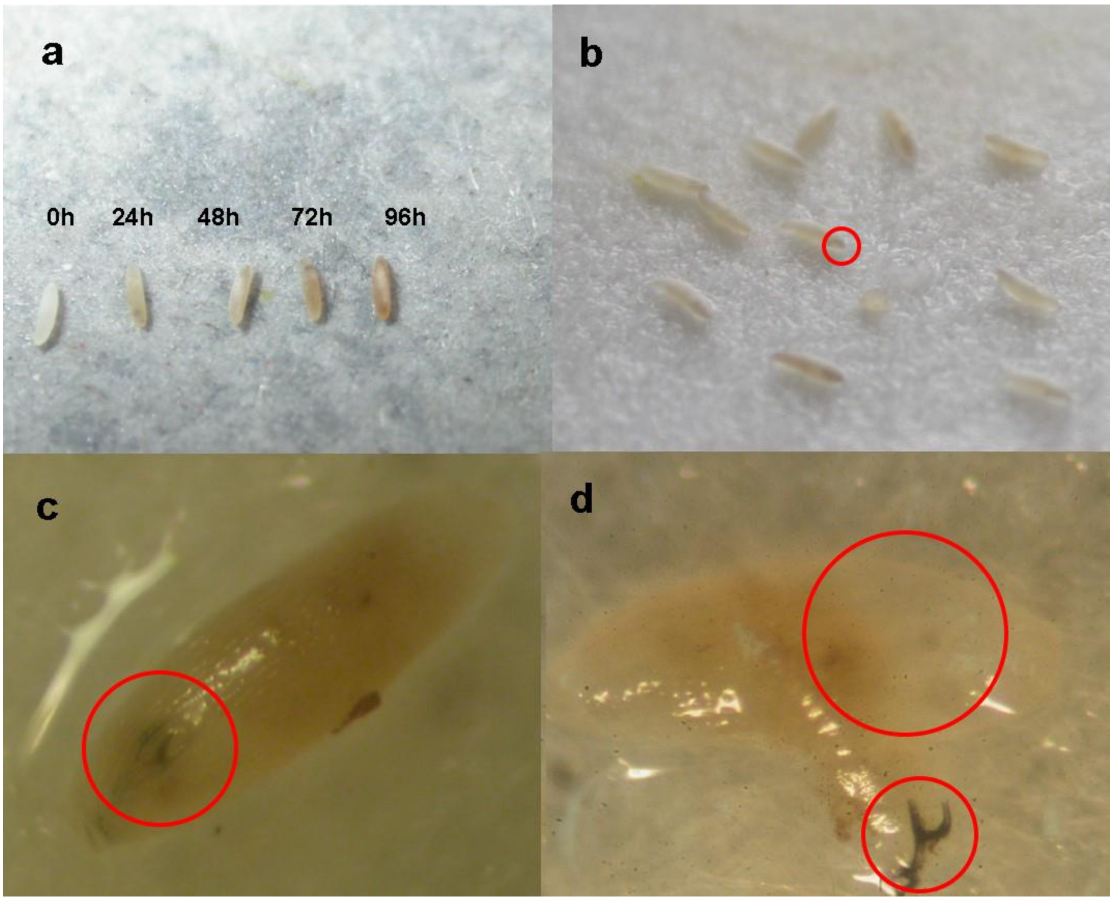

Embryo development in eggs when adult of *Delia antiqua* (Meigen) were treated with lufenuron. a) Embryo development in eggs of treated onion flies with lufenuron at 0h, 24h, 48h, 72h, and 96h after being laid. b) Embryo development in eggs of untreated onion flies. The hooked mouth part could be observed as it was marked in the red circle. c) The hooked mouth part of embryo in eggs. d) The hooked mouth part of embryo and egg shell from unhatched eggs laid by onion flies treated with lufenuron (The egg in Fig S1 d was dissected).

Figure S2 A cylinder-shaped glass chamber used in this paper

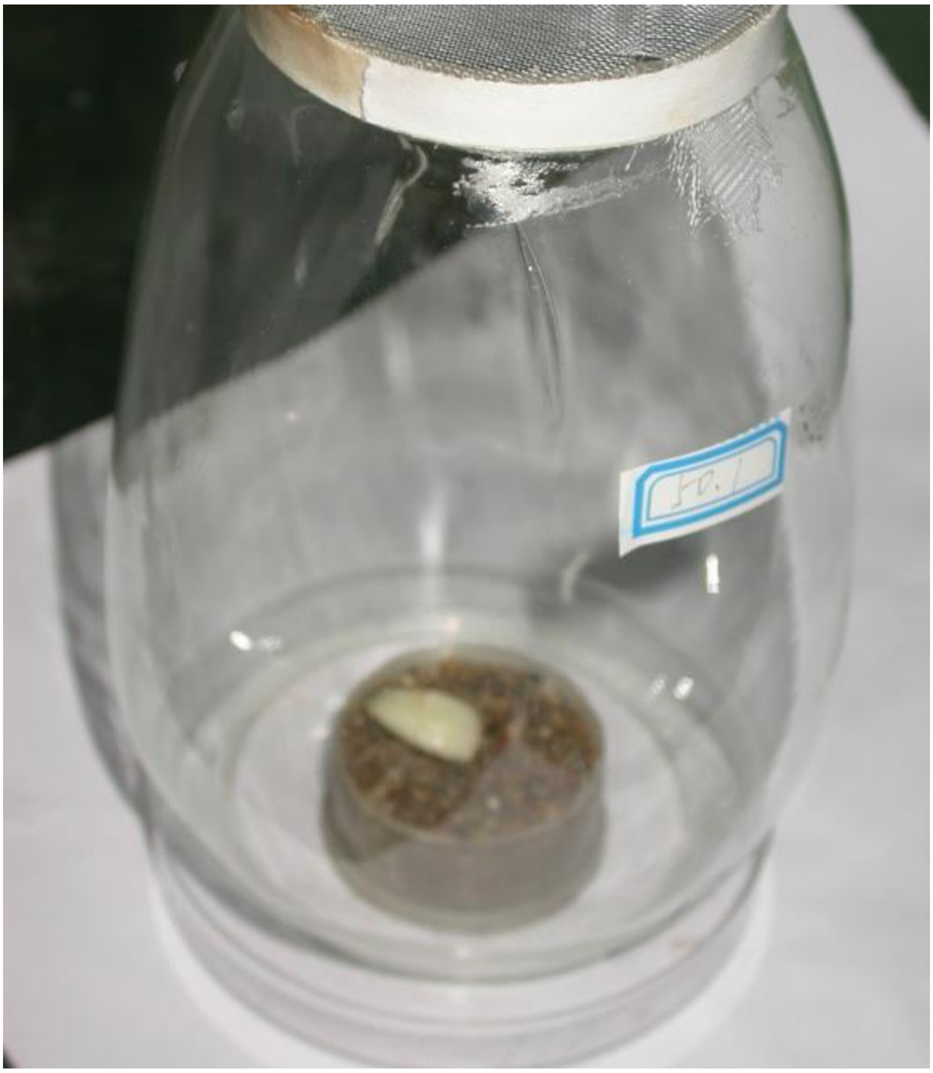

Table S1 Midgut toxicity of 5 insecticides to adults of *Delia antiqua* (Meigen) (72 h)

Insecticide	LC_{50}（95%CL）（mg kg^{-1}）		y=a+bx	
	♀	♂	♀	♂
Clothianidin	3.784（2.799-5.672）	3.146（2.472 -4.125）	y=-1.010+1.747x	y=-1.350+2.712x
Emamectin benzoate	4.505（3.155-7.180）	2.873(1.948-3.931)	y=-0.871+1.333x	y=-0.697+1.521x
Lufenuron	> 2000	> 2000	-	-
Cyromazine	> 2000	> 2000	-	-
Pyriproxyfen	> 2000	> 2000	-	-

Table S2 Preoviposition period of female onion fly treated by selected IGRs.

Treatments	Dose(mg kg^{-1})				*F*	*df*	*P*
	Control	100	500	1000			
Lufenuron	6.86 ±0.38a	6.71 ±0.49a	7.14 ±0.38a	7.00 ±0.58a	1.111	(3,24)	0.364
Pyriproxyfen	6.86 ±0.69bc	6.29 ±0.95c	7.29 ±0.49ab	7.86 ±0.69a	5.909	(3,24)	0.004
Cyromazine	6.71 ±0.95c	7.57 ±0.53c	8.57 ±0.79b	10.43 ±0.79a	29.392	(3,24)	<0.001

Within each line in the table above, values (days; mean ±s.e., n=7) were analyzed with ANOVA, and different letters denoted significant differences (Tukey's HSD test, $p < 0.05$).

Chemical and biological materials

All insect growth regulators (IGRs) used in this article were technical-grade chemicals. Lufenuron [(RS)-1-[2,5-dichloro-4-(1,1,2,3,3,3-hexafluoropropoxy)phenyl]-3-(2,6-difluorobenzoyl)urea], cyromazine [N-Cyclopropyl-1,3,5-triazine-2,4,6-triamine] and pyriproxyfen [4-phenoxyphenyl (RS)-2-(2-pyridyloxy)propyl ether] were provided by the Shandong Institute for the Control of Agrochemicals, Ministry of Agriculture (ICAMA).

D. antiqua adults originally collected in garlic fields in year 2000 in Fanzhen, China. Flies were reared at 21 ± 0.5 ℃, 50-60% RH, on a 16:8 h light: dark photoperiod. Newly emerged adults were placed in 25 × 25 × 25 cm wood-profile cages. Sterile absorbent cotton ball soaked with milk, 5% sucrose water solution and water were put into the cage to provide food for the onion flies. In addition, an ovipositional device, a 30-mm petri dish bottom filled with sand into which a piece of garlic was inserted vertically, was put inside the cage [1]. The sand and the piece of garlic were put into water each day and onion fly eggs would float on surface of water, thus these eggs could be collected with a 400-mesh filter.

The treatment method of onion flies

The treatment procedure of onion flies used in this article is similar with the

method in DPIL, which has been standardized for more than 30 years and has been used to develop WHO standard tests for determining resistance in *M. domestica* [2].

Flies to be used in experiments were obtained from pupae screened by mesh (Sieve size, ∅ = 2 mm). Newly emerged flies were sexed and put into cages respectively, and provided with water, 5% sucrose solution and milk. The flies were starved for 24 h before the test. Lufenuron and pyriproxyfen were dissolved with acetone, and cyromazine, methanol (a few drops of distilled water was added into methanol to promote cyromazine solution in methanol) as its solubility is low in acetone. Granulated sugar was impregnated with insecticide by adding 10 mL of the insecticide in an acetone or methanol solution to 20 g sugar and stirring while the acetone or methanol evaporated. Five pairs of flies were put into a cylinder-shaped glass chamber (open at both ends, L=12 cm, ∅=6 cm, supplementary, Fig. S2 online) with 0.5 g of insecticide-treated granular sugar in a small petri dish as the only food, and access to water. The chamber was covered at both sides with mesh. Each chamber was regarded as one replication. For the control group, only acetone or methanol was use to treat the sugar as no different effects between methanol or acetone treatment were observed (data not shown). The tests were carried out at 21 ±0.5 ℃, 50-60% RH, on a 16:8 h light: dark photoperiod. The insecticide-treated granular sugar was not replaced during the 3-day treatment, and it was replaced with foods after the treatment was over. An ovipositional device was also put into the chamber after treatment (supplementary, Fig. S2 online).

References

1 Harris, M. O., Keller, J. E. & Miller, J. R. Responses to n-dipropyl disulfide by ovipositing onion flies: Effects of concentration and site of release. *J. Chem. Ecol.* **13**, 1261-1277 (1987).

2 Keiding, J. Review of the global status and recent development of insecticide resistance in field populations of the housefly, *Musca domestica* (Diptera: Muscidae). *Bull. Entomol. Res.* **89**, S9-S67 (1999).
